# Partnering faith leaders with community health workers increases utilization of antenatal care and facility delivery services in Ethiopia: A cluster randomized trial

**DOI:** 10.7189/jogh.11.04063

**Published:** 2021-10-30

**Authors:** Brandon L Guthrie, Adino T Tsegaye, Katherine C Rankin, Judd L Walson, Getahun A Alemie

**Affiliations:** 1 Departments of Global Health and Epidemiology, University of Washington, Seattle, WAUSA; 2 Department of Epidemiology & Biostatistics, College of Medicine and Health Sciences, University of Gondar, Gondar, Ethiopia; 3 Department of Global Health, University of Washington, Seattle, Washington, USA; 4 Departments of Global Health, Medicine, Pediatrics, and Epidemiology, University of Washington, Seattle, Washington, USA; 5 College of Medicine and Health Sciences, University of Gondar, Gondar, Ethiopia

## Abstract

**Background:**

Ethiopia and other countries continue to experience high rates of maternal mortality and neonatal deaths. Interventions are needed to increase utilization of antenatal care (ANC) and facility delivery services to improve outcomes.

**Methods:**

A cluster-randomized trial was conducted in the Amhara Region of Ethiopia, with 6 communities randomly assigned to receive the intervention and 12 communities monitored as controls. Intervention teams provided outreach to pregnant women and their families. Registry data were used to measure utilization of services provided at health centers in intervention and control communities.The intervention consisted of trained pairs of community health workers and Ethiopian Orthodox priests who worked together to promote health messages around safe delivery. The pairs visited pregnant women and their families in their homes to provide counseling, discuss concerns, and answer questions about ANC and facility deliveries. Intervention impact was measured using facility-level data on monthly number of ANC visits and facility deliveries at the health centers that served the intervention and control communities. Intervention effect was measured using difference-in-difference analyses estimated by generalized estimating equation models.

**Results:**

During the 12-month intervention period, intervention facilities (n = 6) recorded 14% more ANC1 visits (relative risk RR = 1.14; 95% confidence interval (CI) = 1.09-1.19; *P* < 0.001) and 26% more ANC4 visits (RR = 1.26; 95%CI = 1.18, 1.34; *P* < 0.001) compared to control health centers (n = 12). The intervention health centers experienced a 10% increase in facility deliveries over what would have been expected had the intervention not occurred (RR = 1.10; 95% CI = 1.05-1.16; *P* < 0.001).

**Conclusions:**

Promotion of safe delivery through home visits by community health workers paired with Ethiopian Orthodox priests increased utilization of ANC and facility delivery services. This approach could leverage the influential role of faith leaders and increase the impact of community health workers in Ethiopia.

**Trial registration:**

NCT04039932.

Ethiopia has made great strides in reducing maternal and child mortality in the last decade. Despite these gains, maternal mortality in Ethiopia remains high, with women experiencing a 1 in 55 lifetime risk of maternal mortality (a maternal mortality ratio of 401/100 000 live births) [1-3]. Neonatal mortality also remains high, increasing from 29 to 30 deaths per 1000 live births between 2016 and 2019, although overall child mortality declined (from 67 to 55 deaths per 1000 live births) [4]. Access to care varies widely between urban populations and Ethiopia’s rural populations, which constitute the majority of the country’s citizens.

Several interventions have been demonstrated to reduce maternal and neonatal deaths. These include attendance at antenatal care visits (4 recommended) and delivery in a facility with skilled health care providers. In the Amhara region of Ethiopia, many women do not utilize these services. For example, 17% of women in the Amhara region do not receive any antenatal care and only 51% receive all four recommended visits. In addition, just over half of women (51%) give birth in a health facility with health care providers equipped to provide a safe delivery and manage complications [4].

There are many factors that contribute to low maternal service utilization in Ethiopia, including geographic, economic, cultural, and health system barriers. Ethiopia has rapidly scaled its health system over the past two decades, but still faces shortages of health care workers. The health extension program has been a successful strategy in shifting some primary health care activities to cadres of community health workers, but still faces challenges in covering large catchment areas and disseminating health education. As of 2017, there were only 0.84 nurses and midwives per 1000 people [5], the cadre of health care workers that staff the primary health care system. Given the strained health system and existing strengths of the Health Extension Program, there is a need for improved community engagement strategies that leverage existing community networks, particularly in rural areas, to mobilize the community to improve maternal and child health.

Community mobilization strategies often work to identify community leaders or influencers to promote health education and perform outreach. In particular, faith leaders are frequently identified as community members with the potential to influence societal norms and promote or hinder health behaviors [6]. Prior studies have shown that engagement of faith leaders, particularly for vaccination, malaria, and HIV health campaigns, can improve the engagement of communities in health education and practice [7]. Less is known, however, about models of faith leader engagement for increasing service utilization among women around childbirth, a critical window for preventing maternal and child deaths.

The Amhara region of Ethiopia provides a unique setting to test a model for faith leader engagement, with 84% of the population adhering to the Ethiopian Orthodox Christian (EOC) faith. The EOC maintains a high degree of influence on cultural and behavioral practices with their followers in most communities in this region. We conducted a community randomized trial to test the hypothesis that deploying faith leaders with community health workers in outreach and education activities for maternal health would improve attendance at ANC visits and increase uptake of facility delivery.

## METHODS

### Study setting

**Figure 1:**
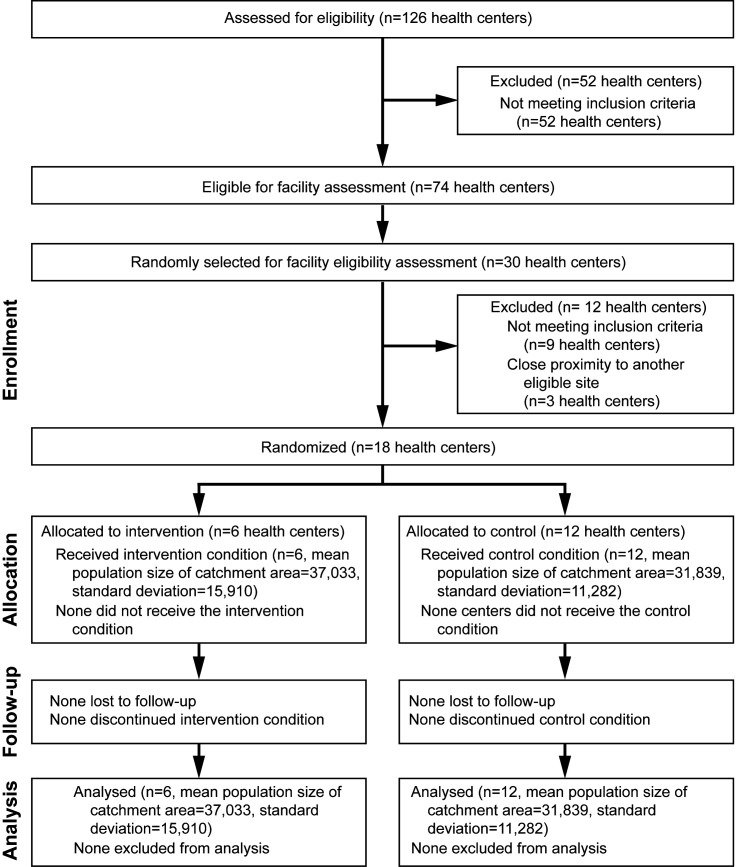
CONSORT diagram of community cluster enrollment, randomization, follow-up and analysis.

The Faith Leaders Advocating for Maternal Empowerment (FLAME) study was a community-based intervention to increase the number of women who utilize antenatal care (ANC) and facility delivery services in the Gondar areas of Ethiopia. The effect of the intervention was assessed based on outcomes extracted from registry data at health centers that serve study communities. Health centers are the lowest level primary health care facilities in the Ethiopian health system where comprehensive maternal and child health services are provided, including standard ANC and skilled delivery. The study was conducted in the North, West, and Central Gondar Zones of the Amhara Region of Ethiopia. Pregnant women and their families in the selected areas are likely to belong to the Ethiopian Orthodox religion, work as farmers, and be of low socio-economic status. Our sample for this intervention included health centers serving rural or peri-urban populations with similar ANC and facility delivery volumes. A total of 18 health centers were randomly selected from 30 eligible health centers in the study area, out of the 126 health centers in the 22 districts constituting the three zones (**Figure 1**). Determination of health center eligibility was based on a systematic assessment of all health centers in the study area. For each selected facility, the catchment area was defined as the population served by the health center. Health centers served as the unit of randomization for the trial and sites were randomized at a ratio of 2 control communities per 1 intervention community.

### Randomization

The health centers selected for the trial were enrolled between August and December 2017. Randomization was conducted by a biostatistical consultant who was not involved in the study after all study sites were enrolled in the trial. Randomization was conducted using a random number generator to assign a random number to each study health center, with the 6 smallest numbers assigned to the intervention group and the largest 12 numbers assigned to the control group. No concealment of allocation was conducted. ATT and GAA enrolled health centers and assigned health centers to study arm based on the randomization list.

### Eligibility criteria

Health centers were eligible for inclusion in the study if they were accessible for data collection, defined as being within 4 hours of Gondar city by car and no more than one-hour walking distance from the road (during the rainy season, some facilities require a walk of up to 4 hours). Eligible health centers had a minimum monthly average of 50 live births, based on their registry reporting to the Federal Ministry of Health. They were required to maintain a maternal child health registry consistent with Federal Ministry of Health standards and had to have adequate capacity to deliver ANC and delivery care, as defined by the Federal Ministry of Health. Eligible health centers also had to serve a catchment population of whom at least 75% identified as Orthodox Christian. Health centers were ineligible if they had >200 deliveries per month or if they were located within an area with ongoing civil unrest and security threats at the time of site selection.

Trial registration: https://clinicaltrials.gov/ct2/show/NCT04039932.

### Intervention

Intervention communities received a one-year package of outreach and education activities completed by trained faith leaders partnered with community health workers from the Health Development Army (HDA) of the Ethiopian health system. Priests of the Ethiopian Orthodox Church and members of the HDA were recruited from selected communities and received training on maternal health topics (with emphasis on ANC and facility delivery services) and community outreach and counseling prior to the intervention. Pairs of faith leaders and HDA members deployed into their communities to conduct outreach to pregnant women and their families at home, in houses of worship, and in other community forums on topics of maternal health including ANC and facility delivery. Priests and HDA members provided referrals to care for pregnant women they encountered in the community and provided counseling to overcome barriers to care for families.

During the first 6 months of the intervention, the study team held monthly check-in meetings with implementation teams to monitor intervention activities. Meetings decreased in frequency during the second 6 months of the study to 1 meeting every two months. Faith leaders and HDA members received a small transportation reimbursement for their travel and time to attend the study check-in meetings (approximately 15 USD). Study team members traveled to the intervention communities for check-in meetings and led group discussions with the faith leaders and members of the HDA. During these meetings, outreach teams reported activities including number of pregnant women identified and referred for ANC and facility delivery, events where health messages were delivered, problems encountered, and potential solutions identified.

### Study outcomes and data collection

The primary outcomes measured for this study were the number of first ANC visits (ANC1), the number of fourth ANC visits (ANC4) and the number of births attended by skilled health personnel at the selected health facilities. These outcomes were measured as monthly counts at each of the study health centers. Data were collected every other month by trained data collectors who abstracted information from the registries maintained at each of the participating health centers. Data collection was monitored by a data manager at the University of Gondar. Twelve months of data were collected from health center registries for the period prior to the start of the intervention, which served as a baseline measure of the outcomes to be used for comparison between control and intervention health centers.

### Statistical analysis

Characteristics of study health centers and their catchment populations were compared between control and intervention health centers based on medians and interquartile ranges. Differences between groups were assessed based on Poisson regression with robust standard errors. The effects of the intervention on the outcomes of interest were estimated based on a difference-in-difference approach that used the baseline values for control and intervention health centers to estimate the counterfactual levels of the outcome during the intervention period among intervention communities had the intervention not occurred [8]. A difference-in-difference approach was used for this analysis to account for any differences between control and intervention health centers during the baseline period and to account for potential temporal trends resulting in different mean levels of the outcome between baseline and intervention periods. These quantities were estimated using generalized estimating equations (GEE) with a Poisson family and a log link, which allowed for the correlation between outcomes measured at the same health centers across time. Effect estimates were expressed as relative risks (RR); a multiplicative effect size estimate was selected because of variation in baseline counts of the outcome of interest, and it was hypothesized *a priori* that the intervention would have an effect that was proportional to the baseline level of the outcomes rather than an additive effect. The models contained a term for the study group (ie, control or intervention), the study period (ie, baseline or intervention period), and an interaction term between the study group and the study period. This model produced an estimate of the mean monthly count of the outcomes across the 12-month baseline and intervention periods, allowing for different means between study groups and study periods, and the difference between study groups was allowed to vary independently between baseline and intervention periods. The difference-in-difference effect estimate was determined by the interaction term in the model. All analyses were conducted in Stata version 14 (Stata Corp, College Station, TX, USA). The analysis plan was defined *a priori* and was registered in clinicaltrials.gov (NCT04039932).

### Power and sample size

The intervention trial included 6 intervention facilities and 12 controls facilities. The trial was designed to have 80% power to detect an 8% increase in the total number of facility deliveries between control and intervention facilities during the trial period, based on a Poisson distribution with α = 0.05 and accounting for clustering by facility assuming a coefficient of variation κ = 0.25, consistent with published values from other studies of MNCH outcomes [9].

### Ethical approval

Ethical approval was granted by the Institutional Review Board (IRB) at the University of Gondar. The study received a waiver of consent for the interactions between the intervention teams and the pregnant women and their families. This waiver was requested because it would not have been feasible to obtain individual consent, and the waiver was granted because the research was judged to be no more than minimal risk, all interactions with pregnant women and their families was voluntary, and no information about individual participants was collected. All outcomes were aggregated at the facility level and thus no identifiable information was accessed or recorded.

### Patient and public involvement

Healthcare workers and members of the public participated in focus group discussions that informed the design of the intervention and implementation of the trial. Representatives of the Ethiopian Orthodox Church were involved in planning of the trial and in identification and training of priests who participated in the trial. Results were disseminated to the Ethiopian Ministry of Health and to representatives of the Ethiopian Orthodox Church.

## RESULTS

We selected 18 health facilities that each serve distinct catchment areas that were eligible for the study intervention. Of these, 6 were randomly assigned to the intervention arm and 12 were assigned to the control arm. The estimated median catchment population was somewhat larger for control facilities (33 158; interquartile range (IQR)  = 26 081 to 36 174) compared to intervention facilities (32 756; IQR = 30 020 to 35 378) (**Table 1**). Facility characteristics were similar between control and intervention sites, although control facilities had modestly higher median staffing levels for clinical officers (2 vs 1), nurses (8 vs 6), and midwifes (3 vs 2), while a somewhat smaller median number of members of the HDA were linked to control facilities as compared to intervention facilities (10 vs 11.5).

**Table 1:** Characteristics of facilities that served control and intervention communities*

	Control facilities (n = 12); median (IQR)	Intervention facilities (n = 6); median (IQR)	*P*-value
Communities (Kebeles) served	5.5 (4, 7)	6.5 (5, 8)	0.25
Catchment population	33 158 (26,081, 36 174)	32 756 (30,020, 35 378)	0.45
Delivery beds	2 (2, 2.5)	2 (2, 3)	0.52
Clinical officers	2 (1, 2.5)	1 (0, 1)	0.13
Nurses	8 (6, 8.5)	6 (6, 7)	0.61
Midwives	3 (3, 4)	2 (2, 3)	**0.01**
Pharmacists	1 (1, 2)	1 (1, 2)	0.39
Laboratory technicians	1 (0, 2)	1 (1, 1)	0.99
Health extension workers	10 (8.5, 13.5)	11.5 (11, 14)	0.21
Health Development Army workers	184 (146, 214)	240 (194, 365)	0.21

*Characterists of intervention and control facilities were compared using Poisson regression models with robust standard errors. Statistically significant differences at the level of α = 0.05 are indicated in bold.

During the 12-month pre-intervention baseline period (8 June 2017 to 7 June 2018), there were no differences between control and intervention facilities in the primary outcome measures. During the baseline period, the mean number of monthly ANC1 visits was 50.1 among control facilities and 50.8 visits among intervention facilities (RR = 1.02; 95% confidence interval (CI)  = 0.92-1.12; *P* = 0.76). The mean monthly number of ANC4 visits was 24.4 among control facilities and 24.6 visits among intervention facilities (RR = 1.01; 95% CI = 0.88-1.16; *P* = 0.91). Control facilities had a mean of 35.2 facility deliveries per month compared to 32.8 deliveries per month at the intervention facilities (RR = 0.93; 95% CI = 0.82, 1.07; *P* = 0.31). Facility-specific patterns of ANC1, ANC4, and facility deliveries during the baseline and intervention periods are shown in Figures S1-S3 in the **Online Supplementary Document**.

### First ANC visits

**Figure 2:**
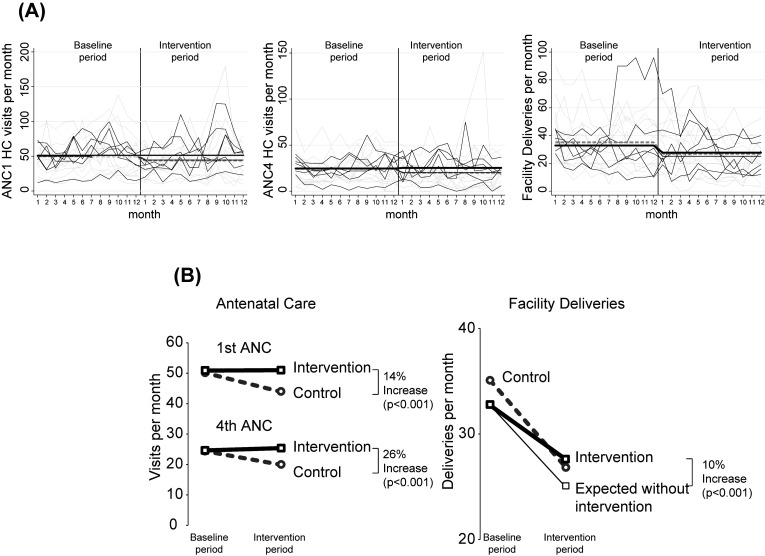
Effect of the faith leader / community health worker partnership intervention on antenatal care engagement and facility deliveries. **Panel A.** Time series of monthly outcomes measured at study health centers comparing intervention and control sites show both between and within facility variation in outcomes over time. Thin black lines indicate intervention facilities and thin grey lines indicate control facilities. The thick black line represents the mean number of outcomes for intervention facilities and the thick grey dashed line represents the mean for control facilities. The medium black line indicates the mean that would have been expected in intervention facilities had the intervention not occurred. The vertical line indicates the timing of the intervention implementation. **Panel B.** Difference-in-difference effect of the intervention.

During the 12-month intervention period (8 June 2018 to 7 June 2019), the mean number of ANC1 visits per month decreased significantly to 44.0 among control facilities (n = 12) (*P* < 0.001) (**Figure 2****,** Panel A), while in the intervention facilities (n = 6), the mean number of monthly ANC1 visits was 50.9, remaining unchanged from the baseline period (*P* = 0.97). Accounting for the pre-intervention baseline levels, the estimated difference-in-difference intervention effect was an increase of 14% in ANC1 visits compared to the estimated counterfactual condition had these communities not received the intervention (RR = 1.14; 95% CI = 1.09-1.19; *P* < 0.001).

### Fourth ANC visits

At control facilities (n = 12) during the intervention period, monthly ANC4 visits decreased to a mean of 20.0 visits compared to the pre-intervention period (*P* < 0.001) (**Figure 2****,** Panel B). In contrast, at intervention facilities (n = 6), mean monthly ANC4 visits increased non-significantly to 25.3 (*P* = 0.24). This shows an intervention effect of a 26% difference-in-difference increase in the number of fourth ANC visits in the intervention communities (RR = 1.26; 95% CI = 1.18, 1.34; *P* < 0.001).

### Facility deliveries

During the intervention period, compared to the pre-intervention period, the mean number of monthly facility deliveries decreased in control facilities (n = 12) to 26.9 per month (*P* < 0.001) and in intervention facilities (n = 6) decreased to 27.7 per month (*P* < 0.001) (**Figure 2****,** Panel B). While facility deliveries decreased in intervention facilities, because the decrease was significantly less than in the control facilities, the difference-in-difference intervention effect on facility deliveries was an increase of 10% in monthly facility deliveries above what would have been expected had the intervention not occurred (RR = 1.10; 95% CI = 1.05-1.16; *P* < 0.001).

### Service disruptions, outliers, and sensitivity analyses

We observed a number of outlier occurrences during the baseline and intervention period due to short-term events that affected utilization of ANC and delivery services at some health facilities. Throughout the intervention period, particularly in the second half of the period, there were intervals of insecurity and political unrest that affected many of the study communities, both control and intervention. During these intervals of insecurity, characterized by civil unrest due to political instability in the country, movement within the affected communities was unsafe, leading to lower service utilization at the affected facilities. These intervals of insecurity were intermittent and affected different areas at different times and to differing degrees. While logs were kept by the study team to note changes in security conditions across sites, it was not possible to collect detailed, systematic information about the exact locations and timing of these intervals of insecurity, and therefore it is not possible to accurately account for their effect in the analysis. However, we know that because of insecurity, control facility #10 was closed for months 7-9 of the intervention period. There was a subsequent spike in ANC1 and ANC4 visits at this facility in month 10 of the intervention period as women who were unable to attend in the previous months came in for missed ANC visits. In sensitivity analyses that excluded facility #10, the intervention effect on ANC1 decreased slightly (RR = 1.12; 95% CI = 1.08-1.17; *P* < 0.001), the effect on ANC4 increased slightly (RR = 1.32; 95% CI = 1.25-1.39; *P* < 0.001), and the effect on facility deliveries decreased slightly (RR = 1.08; 95% CI = 1.02-1.13; *P* = 0.004).

Facility 3, an intervention facility, experienced a period of sharply elevated number of monthly ANC4 visits (35-61 vs 21-30 per month) and facility deliveries (70-96 vs 26-40 per month) starting in month 8 of the baseline period and extending to month 2 of the intervention period for facility deliveries and month 6 for ANC4 visits. After this period, both ANC4 and facility deliveries returned to levels more consistent with the baseline period. Upon investigation, this elevation in service utilization was attributed to a robust health education campaign conducted by the health facility during this period. In sensitivity analyses that excluded facility 3, the intervention effect on ANC4 visits decreased slightly (RR = 1.23; 95% CI = 1.15-1.31; *P* < 0.001) and the effect on facility deliveries increased slightly (RR = 1.13; 95%CI = 1.07, 1.19; *P* < 0.001).

## DISCUSSION

This community cluster-randomized trial showed that utilization of ANC services and facility deliveries were significantly increased by an intervention that paired faith leaders with community health workers to conduct community outreach and education. The faith leaders were Ethiopian Orthodox priests and the community health workers were members of the Health Development Army. The intervention increased ANC1 visits by 14%, ANC4 visits by 26%, and facility deliveries by 10% compared to what would have been expected had the intervention not occurred, which was estimated based on the pre-intervention baseline period and the experience of control facilities during the baseline and intervention periods. The intervention had the greatest impact on increasing the number of ANC4 visits, which we believe is consistent with a mechanism of action involving increased recognition of the importance of completing the full course of recommended ANC visits, leading to greater motivation to participate in ANC. Attendance at a first ANC visit is relatively common in this area, estimated at 83% of pregnancies, although women in rural areas have lower ANC utilization [10]. This provided a limited opportunity for the intervention to increase ANC1 participation. However, many of these women do not complete the recommended 4 ANC visits (51%) [10]. Therefore, we believe that this intervention was particularly effective in encouraging women to complete all of their ANC visits. Additional qualitative research would provide insight into which elements of the intervention were most motivating for women and their families. While the intervention did not increase facility deliveries relative to the baseline period, the difference-in-difference analysis indicates that the intervention had the effect of preventing the magnitude of decline in facility deliveries observed at control health centers during the intervention period, which were possibly due to the effects of local insecurity. The intervention’s lower magnitude of effect on facility deliveries may reflect larger structural barriers to facility delivery that cannot be readily overcome through motivational counseling and education, highlighting opportunities to identify specific barriers that may be addressable through additions to the intervention package, such as strategies to overcome transportation barriers or childcare concerns. These results are likely most generalizable to Ethiopian Orthodox communities throughout Ethiopia, but other faith communities that have leaders who serve important influential roles in the community, most notably Muslim communities, would likely benefit from a similar intervention. We believe that the intervention may be generalizable to other settings, but would likely depend on the presence of engaged community leaders who are willing and able to partner with community health workers.

Most evaluations of the scope and effectiveness of faith-based organizations in relation to health have focused on faith-based health providers (FBHP) that provide direct medical and health services, with much less attention given to the role of faith leaders as influential members of the community who can work in collaboration with health care workers [11], but it is clear that there is an important interface with cultural and faith traditions when it comes to an individual’s health decisions [12] and there are examples of successful partnerships between faith-based organizations and health systems [13]. Despite concerns about quality of services delivered by FBHP, empirical measurements of patients’ perceptions of care provided in these settings are generally positive [14], although concerns have been raised about the effect of theology on provision of sexual and reproductive health care [15]. In the model evaluated in the current study, we show that faith-leaders can work effectively with health centers and community health workers, including in the context of pregnancy and delivery. Our experience is consistent with other findings that partnerships are productive when there is a shared collaborative domain that is not the “sovereign space” of either partner and that these partnerships do not require that the “parties buying into every possible implication of the collaboration” [16]. Our intervention using faith leader/community health worker pairs compares well against other interventions to increase ANC utilization and facility deliveries. A systematic review found some evidence that mHealth interventions may increase attendance at 4 ANC visits by around 10%, compared to 26% in the current study [17]. A systematic review and meta-analysis found evidence of a modest effect of a single community-based intervention (media campaigns, education, or financial incentives for pregnant women) to improve attendance of at least 1 ANC visit (pooled OR = 1.68; 95% CI = 1.02- 2.79), attendance of at least 4 ANC visits (pooled OR = 1.11; 95% CI = 1.01-1.22), and facility deliveries (pooled OR = 1.08; 95% CI = 1.02-1.15) [note that ORs will be larger than equivalent RRs because these outcomes are not rare]. Experience from other successful models of partnerships between faith communitees and the health system have identified important guiding principles for longterm success and sustainability that include the assessment of the asset base, focusing on community scale implementation, building trust with the community, leading with humility, following community-based participatory research principles, taking a person-centric rather than hospital-centric perspective, using an integrative strategy that blends traditional clinical or biomedical care with community caregiving, and using a shared-data protocol that includes indicators of value to all stakeholders [6]. Additional research will be needed to evaluate the broader implementation, fidelity, sustainability, and cost of the intervention that takes these guidelines into consideration.

The intermittent episodes of insecurity that occurred during the intervention period had a considerable impact on the ability of people living in the study communities to travel, which reduced access to the study facilities and reduced utilization of ANC and delivery services. We observed significant decreases in ANC1, ANC4, and facility deliveries at the control facilities during the intervention period compared to the baseline period, and we believe the insecurity issues were at least partly responsible for this decreased utilization. By contrast, ANC1 and ANC4 visits remained essentially unchanged at intervention facilities during the intervention period while facility deliveries declined, but to a lesser degree than at control facilities. This background effect on the outcomes during the intervention period complicates the interpretation of the intervention’s effectiveness. We outline 3 scenarios that could explain the observed results. (Scenario 1) The intervention was effective, and was of a magnitude that offset the decline in ANC visits that would have happened, and it softened the effect that insecurity had on facility deliveries. Under this scenario, in the absence of the insecurity, the intervention would have had an equivalent effect, resulting in an increase in all outcomes. (Scenario 2) The intervention was effective, but its effect was only in lessening the impact of the insecurity, and in the absence of the insecurity, the intervention would not have been effective. (Scenario 3) The intervention was not effective, and the apparent effectiveness was due only to the fact that control communities, due to random chance, happened to have been affected more severely by the insecurity than intervention communities. We believe that the fact that facility deliveries declined at intervention facilities during the baseline period while ANC visits held steady indicates that Scenario 3 is unlikely due to the belief that in the presence of intermittent insecurity, women who were motivated through the intervention to access ANC would have been able to time their ANC visits to coincide with periods of relative safety. By contrast, facility deliveries would be more affected because of the inflexibility of timing. We are unable to determine whether Scenario 1 or 2 is more likely, but this illustrates the benefit of the inclusion of both a baseline period and of control facilities. The difference-in-difference approach enabled us to estimate the intervention effect relative to the expected counterfactual outcome had the intervention not occurred.

### Limitations

This study benefited from a study design that provided a degree of robustness to the unexpected insecurity issues that affected the intervention period and could have obscured the intervention effect in an uncontrolled pre-post study design. However, in addition to the potential that the insecurity affected control and intervention communities disproportionately, as described above, a number other factors limit the interpretation of our results. There were a relatively small number of communities included in this study, and only 6 communities were assigned to the intervention. While this sample size provided sufficiently power to detect the intervention effect, the small number communities increased the chance that confounding factors were not equally distributed between groups. The comparability of facility characteristics between groups and the lack of difference in the number of primary outcomes during the pre-intervention baseline period provides some evidence in support of the success of randomization. The difference-in-difference design was used to account for pre-intervention difference between control and intervention facilities. Outcomes were measured at the facility-level rather than at the level of individuals who were exposed or unexposed to the intervention; therefore the effect estimates should be considered a community-level measure of effectiveness rather than an individual-level measure of efficacy among those who actually received the intervention. Finally, since we relied on facility registries for outcome measurements, it is possible that there was some measurement error in counting outcomes. Any such measurement error is likely to be non-differential in relation to randomization group, and thus the average effect of mismeasurement would be an underestimate of the true intervention effect.

## CONCLUSIONS

This trial demonstrated the feasibility and effectiveness of partnering faith leaders with community health workers at a community level to improve engagement in antenatal care services and facility deliveries attended by skilled providers. Effective training and supervision of these teams is likely to be critically important to scaling and maintaining such an intervention, but this approach holds great potential to improve maternal and child health. We believe that this strategy for community engagement is adaptable to other faith communities and to other types of trusted and influential community leaders in a variety of regions and countries. Further study of the applicability of this model is needed.

## Additional material


Online Supplementary Document
