# Supplementary material for: Partnering faith leaders with community health workers increases utilization of antenatal care and facility delivery services in Ethiopia: A cluster randomized trial
Source: J Glob Health. 2021 Oct 30;11:04063. doi: 10.7189/jogh.11.04063 (PMC8564884; doi:10.7189/jogh.11.04063)
Supplement: Online Supplementary Document [file jogh-11-04063-s001.pdf]

Online Supplementary Documents

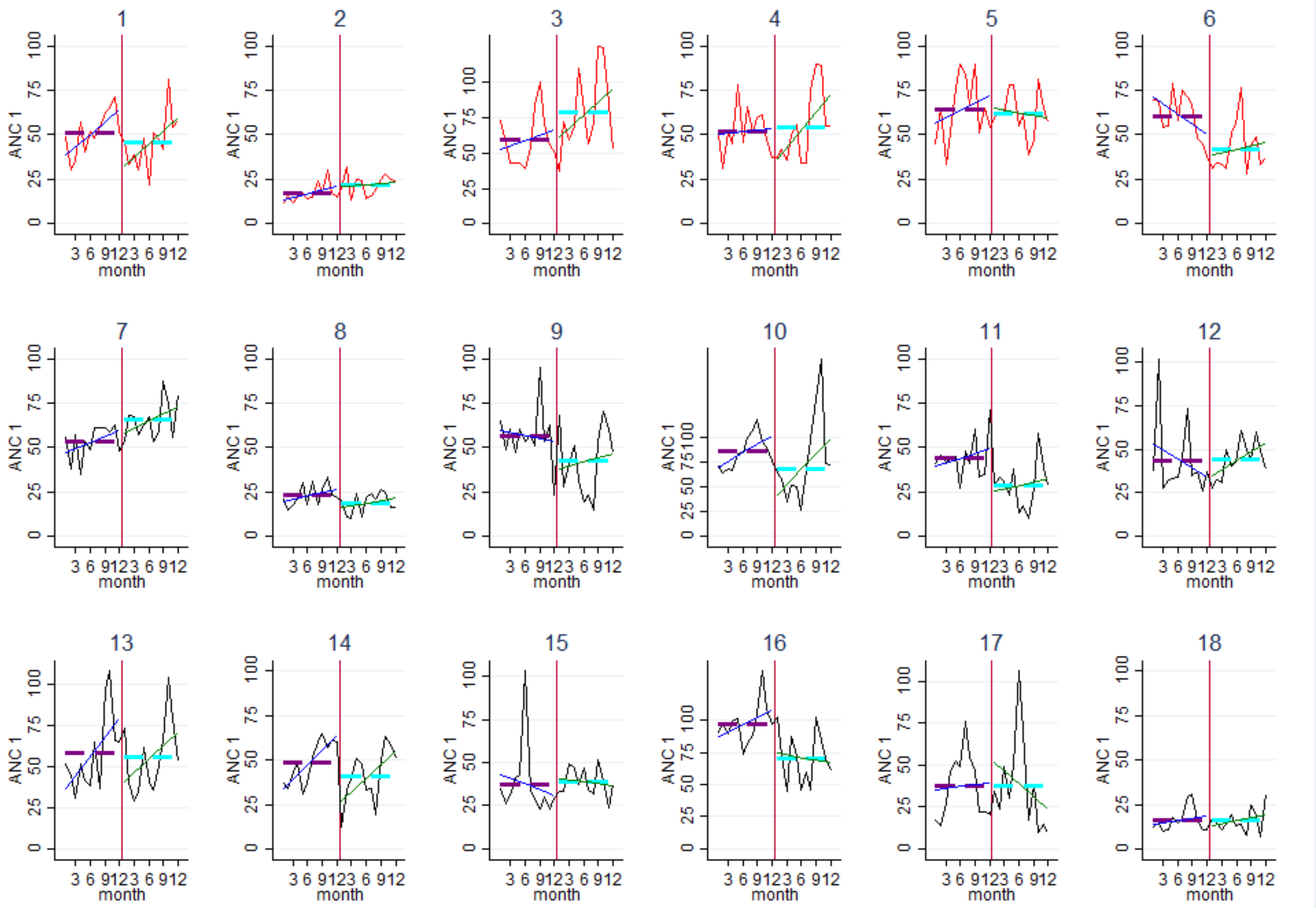

Figure S1. Facility-specific monthly number of first ANC visits. Sites 1-6 were intervention health centers and 7-18 were control health centers.

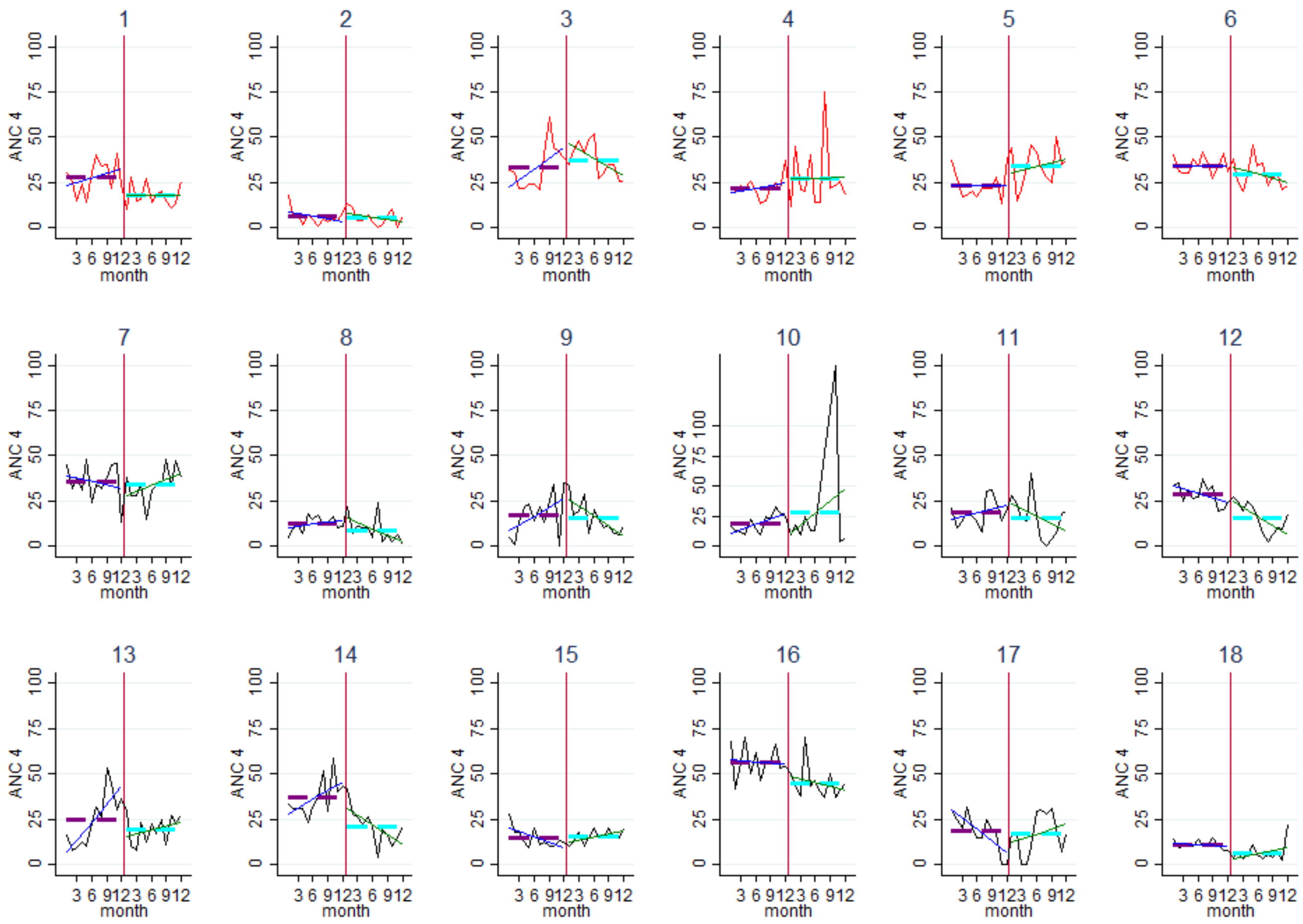

Figure S2. Facility-specific monthly number of fourth ANC visits. Sites 1-6 were intervention health centers and 7-18 were control health centers.

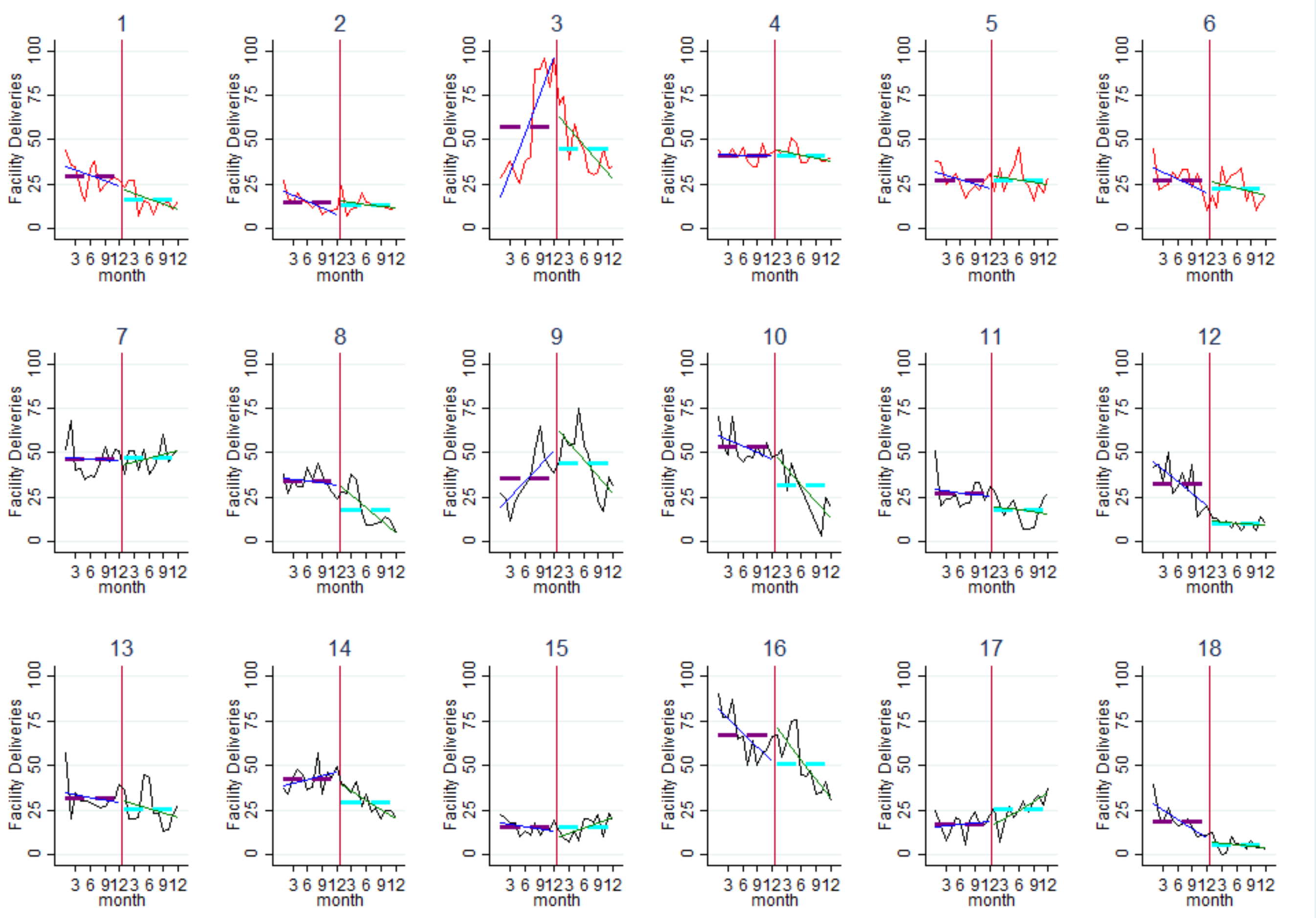

Figure S3. Facility-specific monthly number of facility deliveries. Sites 1-6 were intervention health centers and 7-18 were control health centers.
